# Transcriptomic Assay of CD8^+^ T Cells in Treatment-Naïve HIV, HCV-Mono-Infected and HIV/HCV-Co-Infected Chinese

**DOI:** 10.1371/journal.pone.0045200

**Published:** 2012-09-13

**Authors:** Jin Zhao, Lina Yi, Jing Lu, Zheng-Rong Yang, Ying Chen, Chenli Zheng, Dan Huang, Yu-Feng Li, Lin Chen, Jinquan Cheng, Hsiang-fu Kung, Ming-Liang He

**Affiliations:** 1 Stanley Ho Center for Emerging Infectious Diseases, Faculty of Medicine, The Chinese University of Hong Kong, Hong Kong, China; 2 Shenzhen Center for Disease Control and Prevention, Shenzhen, China; 3 College of Bioengineering, Xihua University, Chengdu, China; 4 Li Ka Shing Institute of Health Sciences, Faculty of Medicine, The Chinese University of Hong Kong, Hong Kong, China; National Institute of Infectious Diseases, Japan

## Abstract

**Background:**

Co-infection with HIV and HCV is very common. It is estimated that over 5 million people are co-infected with HIV and HCV worldwide. Accumulated evidence shows that each virus alters the course of infection of the other one. CD8+ T cells play a crucial role in the eradication of viruses and infected target cells. To the best of our knowledge, no one has investigated the gene expression profiles in HIV/HCV-co-infected individuals.

**Methodology:**

Genome-wide transcriptomes of CD8+ T cells from HIV/HCV-co-infected or mono-infected treatment-naïve individuals were analyzed by microarray assays. Pairwise comparisons were performed and differentially expressed genes were identified followed by quantitative real-time PCR (qRT-PCR) validation. Directed Acyclic Graphs (DAG) from Web-based Gene SeT AnaLysis Toolkit (WebGestalt) and DAVID bioinformatics resources 6.7 (the Database for Annotation, Visualization, and Integrated Discovery) were used to discover the Gene Ontology (GO) categories with significantly enriched gene numbers. The enriched Kyoto Encyclopedia of Genes and Genomes (KEGG) pathways were also obtained by using WebGestalt software.

**Results and Conclusions:**

A total of 110, 24 and 72 transcript IDs were shown to be differentially expressed (> 2-fold and *p*<0.05) in comparisons between HCV- and HIV-mono-infected groups, HIV/HCV-co-infected and HIV-mono-infected groups, and HIV/HCV-co-infected and HCV-mono-infected groups, respectively. In qRT-PCR assay, most of the genes showed similar expressing profiles with the observation in microarray assays. Further analysis revealed that genes involved in cell proliferation, differentiation, transcriptional regulation and cytokine responses were significantly altered. These data offer new insights into HIV/HCV co-infections, and may help to identify new markers for the management and treatment of HIV/HCV co-infections.

## Introduction

The human immunodeficiency virus (HIV) and the hepatitis C virus (HCV) are major threats to public health. Presumably, because of the similar transmission routes (*e.g*., drug injection), co-infection with HIV and HCV is very common [1], [2]. It is estimated that over 5 million people worldwide live with chronic HIV/HCV co-infection. However, the prevalence of HIV/HCV co-infection largely varies in different risk groups, with an especially high rate among HIV-positive injective drug users (IDUs) [3]. In China, over 90% of HIV-positive IDUs are co-infected with HCV [1].

Several studies have explored the viral interactions between HCV and HIV infection. However, little is known either about the molecular basis of the interactions or the consequences of host responses. It has been shown that HIV co-infection significantly affected the natural history of hepatic fibrosis in HCV-infected persons[4]. Obviously, a concomitant HIV infection increased the evolution of HCV quasispecies, the level of viremia, and the extrahepatic viral reservoirs [5], [6], [7]. These factors decreased the effects of anti-HCV treatment. Cytokines (IFN-γ, TNF-α and IL-4, etc.) in peripheral blood mononuclear cells (PBMC) and dehydroepiandrosterone sulphate in plasma were greatly decreased in HIV/HCV-co-infected patients [8], [9]. In addition, HIV viral loads displayed additive effects on HCV-triggered mitochondrial DNA depletion, which seems to be associated with the nucleoside analogs' toxicities [10], [11]. However, the impact of HCV infection on HIV/AIDS was not conclusive, even controversy [12], [13]. Some evidence suggests that the increased HCV viral load was associated with AIDS progression. HCV seems to impair the immune reconstitution, leading to an increased rate of AIDS-related death [14], [15]. In other studies, however, HCV did not show any significant effect on HIV-associated disease [16].

Although highly active antiretroviral treatment (HAART) is very successful for treating HIV mono-infection, its impact on HCV has been debated [17], [18]. Sulkowski *et al*. showed that the co-infected individuals experienced an increased risk of hepatotoxicity after HAART [19]. Similarly, the toxicities of interferon-based HCV therapies were also exacerbated in HIV/HCV-co-infected patients with a higher rate of relapses [20], [21]. Therefore, the management of HIV/HCV-co-infected patients is still a major challenge. The underlying mechanisms of highly increased mortality remain elusive.

CD8^+^ T cells play a crucial role in the eradication of viruses and the infected target cells. It has been shown that the high viral load of HCV in HIV/HCV-co-infected patients was associated with profound defects in HCV-specific CD8^+^ T cell responses [22]; however, the gene expression profiles have been monitored only in the total T cells or in the liver biopsy in HIV- or HCV-mono-infected patients [23], [24]. To the best of our knowledge, no-one has reported the global gene expression profiles in HIV/HCV-co-infected individuals. In this study, we investigated the global gene expression patterns of CD8^+^ T cells in the HIV/HCV-co-infected and the HIV- or HCV-mono-infected treatment-naïve individuals without symptoms of liver disease and AIDS.

## Results

### Participants' profiles

Twenty-four HIV-infected, 24 HCV-infected, 24 HIV/HCV-co-infected and 24 non-infected antiretroviral therapy-naïve Chinese (male) were recruited in this study. The status of HCV, HIV, and HBV was strictly monitored in accordance with the stringent guidelines of The Center of Disease Control and Prevention of China (CDC, China). None of the participants showed baseline drug-resistant mutations. The average ages were similar in all the study groups (29.5, 33.0, 31.4 and 27.5 years for the HIV-mono-infected group, the HCV-mono-infected group, the HIV/HCV-co-infected group, and the non-infected group, respectively). All participants are males with no evidence of CD4+ T-cell decline (≥300 cells/ul). There was also no statistically significant difference among their CD8 counts (HIV infections – 1,062±211; HCV infections – 873±412; and HIV/HCV co-infections – 1,237±494, *p*>0.5).

### Analyses of differentially expressed genes

Microarray assays were performed for all three study groups (HIV, HCV-mono-infected, and HIV/HCV-co-infected individuals), and the raw data has been deposited in the ArrayExpress database (access number: E-MEXP-3635). Pairwise comparisons between the three study groups (HCV-mono *vs* HIV-mono, HIV/HCV-co-infected *vs* HCV-mono, and HIV/HCV-co-infected *vs* HIV-mono) were carried out; the numbers of differentially expressed (DE) transcript identifiers (ID) (≥2-fold change, p<0.05) were identified and shown in Table 1. The differentially expressed IDs and their according fold changes were listed in Supplementary Table S1.

**Table 1:** Number of differentially expressed transcript identifiers in pairwise comparisons for CD8+ T cells (fold change >2 and P<0.05).

Differentially expressed transcript identifiers	HCV-mono versus HIV-mono	HCV/HIV-co-infected versus HCV-mono	HCV/HIV-co-infected versus HIV-mono
Up	48	16	2
Down	62	56	21

In total, 28,869 genes were scanned. For comparisons between HCV- and HIV/HCV-infections, most of the identified genes (47/55) showed much lower expressions in co-infections than in HCV mono-infections. This was in sharp contrast to the comparison between the HIV and the HCV mono-infected groups. The latter comparison showed that half (40/81) of the DE genes were downexpressed in the HIV-infected group compared to the HCV-infected group. Results showed high similar expression profiles between HIV infections and HIV/HCV co-infections. Only 24 transcript IDs showed significant changes (≥2-fold); and 4 were identified genes with known functions.

**Figure 1:**
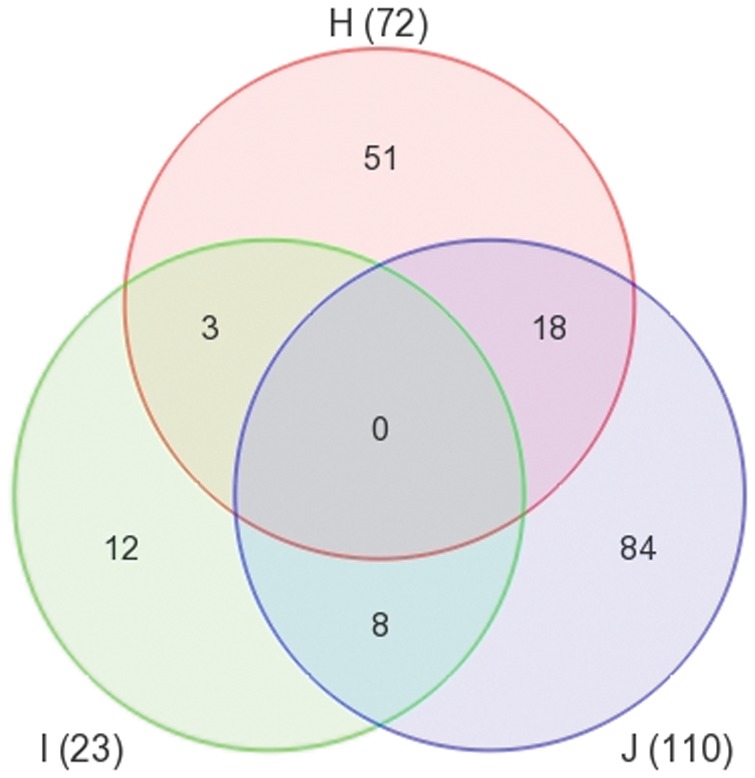
Venn diagram for differentially expressed transcript identifiers in paired comparisons. J: differentially expressed transcript identifiers identified in HCV-mono- *versus* HIV-mono-infected group; H: differentially expressed transcript identifiers identified in HCV/HIV-co-infected *versus* HCV-mono-infected group; I: differentially expressed transcript identifiers identified in HCV/HIV-co-infected *versus* HIV-mono-infected group.

To further inspect the gene expression relationship among the three groups, a Venn diagram was drawn. The circles in Figure 1 represent differentially expressed transcript IDs identified in the three paired comparisons (J – HCV-mono *vs* HIV-mono; H – HIV/HCV-co-infected *vs* HCV-mono; I – HIV/HCV-co-infected *vs* HIV-mono). No genes showed the same pattern of differential expression among the three groups. Eighteen IDs (17 genes, 1 transcript IDs) shared between circle H and J referred to genes which had ≥2 expression fold changes both in the HIV mono-infected and the HIV/HCV co-infected group as compared to the HCV mono-infected group. In the case of HIV mono-infection, gene GPR15 together with 7 other transcript IDs were found to be differently expressed when compared to the HCV mono-infections or to the HIV/HCV co-infections. When we compared the gene expression profiles of the co-infected participants to the mono-infected ones, 3 IDs (1 gene, 2 IDs) were unravelled to display the similar expression changes. The only ID with known function was P2RY13, which showed lower expression levels in the co-infection groups than in the mono-infected groups.

### Functional annotation and biological pathway analysis

To gain insight into the biological significance, the DE genes indentified in each comparison were submitted to functional annotation analysis using DAVID and WebGestalt softwares. Table S2 illustrated all the significant enriched gene categories (Ease scores<0.05) for the DE genes. Aside from the functional annotation classification of DE genes, significant signaling pathways using the Kyoto Encylopedia of Genes and Genomes (KEGG) database was also identified (Table 2). The main attention was paid to the important genes related to in HCV, HIV infections, particularly, the gene ontology (GO) categories involved in immune response, cell cycle, apoptosis and cell migration.

**Table 2:** Kyoto Encyclopedia of Genes and Genomes (KEGG) pathways enriched in pairwise comparisons for CD8+ T cells (P<0.01).

Pairwise comparison	KEGG pathways	Genes(n)	Ratio ofenrichment	P value
HCV-monoversusHIV-mono	Cytokine-cytokine receptor interaction	4	9.06	0.008
HCV/HIV-co-infectedversusHCV-mono	Cytokine-cytokine receptor interactionCell adhesion molecules (CAMs)LysosomeChemokine signalingAntigen processing and presentationAsthmaAllograft rejectionTryptophan metabolismPrimary immunodeficiencyLysine degradationIntestinal immune network for IgA productionAutoimmune thyroid diseaseViral myocarditisApoptosisHematopoietic cell lineageT cell receptor signaling pathway	8544322222222222	25.6331.9229.2518.0128.8457.0345.0242.7748.8838.8834.2232.2823.4419.4419.4415.84	0.0000.0000.0000.0000.0010.0020.0020.0020.0020.0020.0030.0030.0050.0060.0060.009
HCV/HIV-co-infectedversusHIV-mono	None	None	None	None

For genes differently affected by HCV and HIV infection, the functional annotation analyses identified several gene categories associated with cell cycle progress. Among these 81 DE genes, 11 were classified into gene ontology of cell cycle. Compared to the HIV-infected groups, the majority of these genes (9/11) showed lower expression levels in the HCV-infected groups. Genes including TPX2, CCNA2, KIF11, WEE1, ASPM, MKI67 and NUSAP1 were all functioned mainly in the M phase of cell cycle. The different expressions of these genes may reflect un-controlled cell cycle induced by viral infection. We also observed sets of gene categories involved in chromosome organization. Similarly, these genes were all down-regulated in HCV infections. Through modification of chromatin assembly or disassembly, they may also involve in cell cycle progress. Besides, several gene categories involving in cell migration were also identified. The expression levels of these genes were higher in HCV infections than in HIV infections, which may promote the traffick of activated immune effector cells from the periphery to the liver in HCV infected patients. Another important finding is that genes involved in cell proliferation and death were significantly changed, such as TNFRSF9, HIPK2, CD38. Analysis of the DE genes by the KEGG pathways classification showed that pathway of cytokine-cytokine receptor interaction was significantly enriched (Table 2). Genes including CXCL16, TNFRSF9, CCR4 and CX3CR1 contribute most to the enrichment value.

Functional analysis was then performed on the 55 DE genes (17 transcript IDs without known functions) identified in comparisons between HIV/HCV co-infections and the HCV mono-infections. Many of them play crucial roles in immune system development and immune responses. As described above, most of the genes were down-regulated in co-infections when compared to HCV mono-infections. This phenomenon may indicate a suppressed immune response in co-infected individuals, which then would contribute to the accelerated disease progression. Further inspection identified that genes involved in both the innate immune response and the adaptive immune response expressed differently between these two groups. Among the categories of innate immune response, genes especially response to interferon-gamma were identified. These include CXCL16, SLC11A1 and KYNV. While for adaptive immune response categories, genes such as CD86, CD40LG and VCAM1 which functions in lymphocyte activation, proliferation and differentiation were identified. Gene categories associated with behavior, chemotaxis and response to wounding shared a number of genes, including a variety of chemokine genes (CCR4, CX3CR1, CXCL16, and PPBP). They are involved in the recruitment of circulating immune cells to the liver. Besides, the process of antigen processing and presentation were also affected in co-infection groups. This was characterized by the low expression levels of FCER1G, IFI30, APP and CD302. We also observed a lot of genes associated with cytokine production. Most of these genes could regulate interleukin-2 production, a mark for the poly-functional T-cells. Consistently, KEGG pathways analysis showed that genes involved in cytokine-cytokine receptor interaction, cell adhesion molecules (CAMs), lysosome, chemokine signaling pathway, antigen processing/presentation displayed differential expression in co-infected groups as compared to the HCV mono-infected group (Table 2).

In total, 4 (1 upregulated, 3 downregulated) DE genes were identified in comparisons between HIV/HCV co-infection groups and HIV mono-infection groups. According to the functional annotation, 3 of them (P2RY13, ENTPD1 and GPR15) were assigned to GO categories of G-protein coupled receptor protein signaling pathway (GO: 0007186) and functioned in receptor mediated immune response. Interestingly, we found that OCLN, which regulates the permeability barrier function of the tight junction, was significantly downregulated. Consistently, it has been reported that the OCLN expression was also decreased in HIV Tat protein transfected cells. The further down-regulation of OCLN may associate with HIV disease progressions in HIV/HCV co-infected patient. No significant KEGG pathways were identified between the two groups.

### Validation of differentially expressed genes

**Figure 2:**
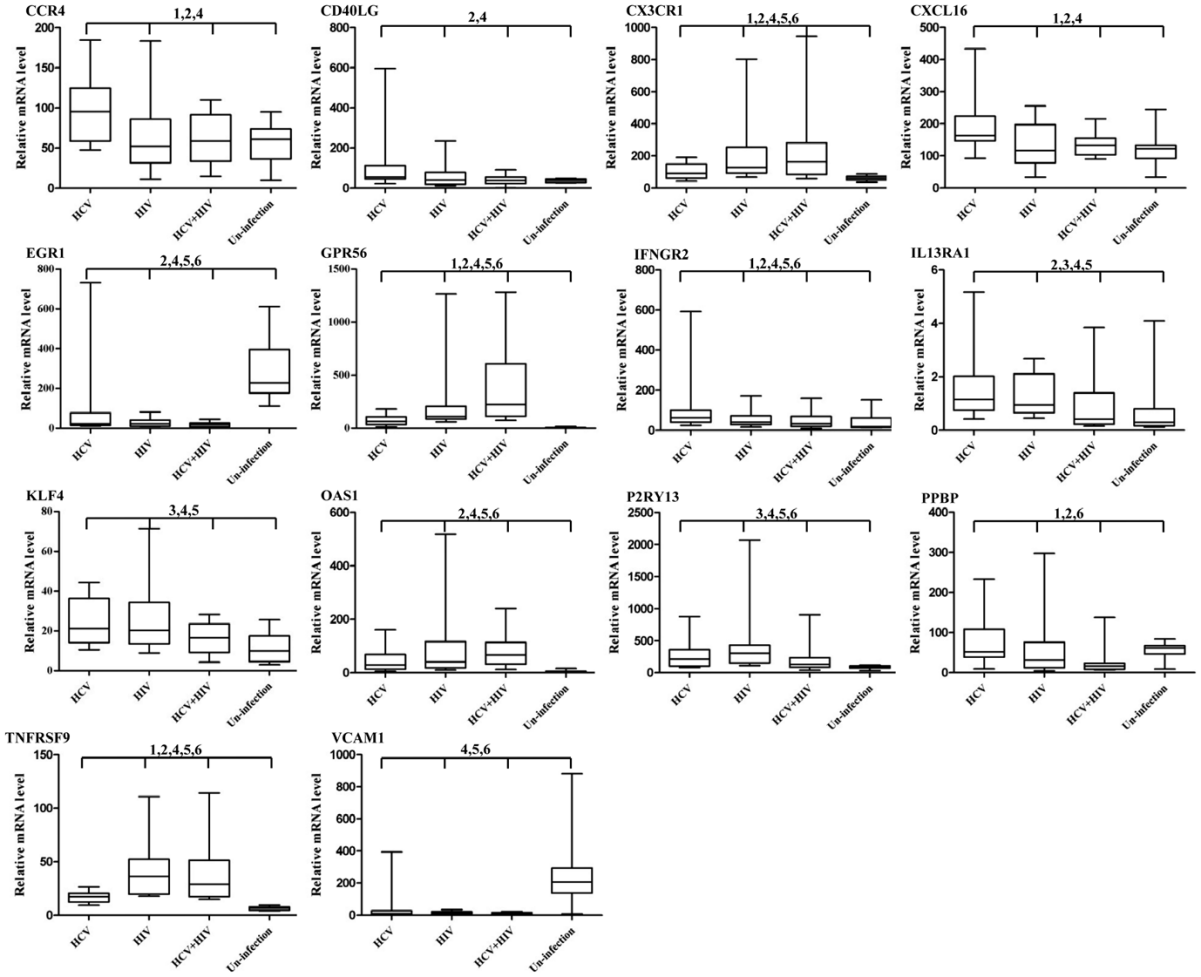
Validations of differentially expressed genes by quantitative real-time PCR. The mRNA levels of selected genes were measured in HCV-mono-infected, HIV-mono-infected, HIV/HCV-co-infected and uninfected individuals by quantitative real-time PCR. The relative mRNA value was calculated by the following formula: R = 2^CT (GAPDH-X)^*10^3^, where X represents the CT value of each gene. Box-plot illustrated the medians with 25% and 75%. 1–6 represent comparisons between HCV infection and HIV infection, HCV and HIV/HCV co-infection, HIV and HIV/HCV co-infection, HCV infection and uninfection, HIV infection and uninfection, and HIV/HCV co-infection and uninfection with *p*<0.05, respectively.

To confirm the observations from the microarray analysis, the mRNA levels of the selected DE genes were measured by quantitative real-time PCR (qRT-PCR). The DE genes in KEGG pathway of cytokine-cytokine receptor interaction were selected for qRT-PCR confirmation; this is because this pathway was identified as being enriched in comparison between HIV and HCV mono-infections (R = 9.06, 4 genes: CXCL16; TNFRSF9; CCR4; and CX3CR1), as well as in comparison between HIV/HCV co-infections and the HCV mono-infections (R = 25, 8 genes: CXCL16; TNFRSF9; CCR4; CX3CR1; IL13RA1; IFNGR2; CD40LG; and PPBP). In addition, the DE genes not involved in any enriched KEGG pathway were randomly selected. In the case of HIV *vs* HCV mono-infections, OAS1 and VCAM1 were selected. EGR1, KLF4, and GPR56 were selected in the case of HIV/HCV co-infection *vs* the HCV mono-infection. P2RY13 was selected to represent HIV/HCV co-infection *vs* the HCV mono-infection cases. The mRNA isolated from CD8^+^ T cells of each individual was used for qRT-PCR experiments. The expression levels of each gene in all four groups were evaluated (Figure 2) and compared to the results obtained from microarray assays (Table 3). The qRT-PCR results showed that the expressing profiles of 10 genes (CCR4; CD40LG; CX3CR1; CXCL16; IFNGR2; IL13RA1; EGR1; KLF4; GPR56; and TNFRSF9) exactly matched with the observation in microarray assays; another three genes (PPBP; OAS1; and P2RY13) matched at least one express pattern out of three pairwise comparisons. Only the VCAM1's expression pattern was not in concordance with the microarray data. These results largely confirmed the data from the microarray assays. Besides, the mRNA levels of all the selected genes in un-infected persons with matched ages were also analyzed. As shown in Figure 2 and Table 3, the expression levels of CX3CR1, GPR56, OAS1, P2RY13 and TNFRSF9 in infected groups elevated significantly as compared to the uninfected healthy group; while the expression levels of EGR1 and VCAM1 decreased in the infected groups. Three genes: CCR4, CD40LG and CXCL16 only changed in HCV infections. For genes such as IFNGR2, KLF4 and IL13RA1, both the HCV and HIV infection could induce high expression; however no significant difference was detected in comparisons between co-infections and un-infections. Still there is a gene (PPBP) only showing altered expressions in co-infections.

**Table 3:** Quantitative real-time PCR validations of differentially expressed genes (*P*<0.05).

Gene symbol	Method	HIV + HCV *vs* HCV(fold-change)	HIV + HCV *vs* HIV(fold-change)	HCV *vs* HIV(fold-change)	HCV *vs* Uninfection (fold-change)	HIV *vs* Uninfection(fold-change)	HCV + HIV *vs* Uninfection(fold-change)
CCR4	MA	−2.51	NS	2.59	ND	ND	ND
	qPCR	−1.63	NS	1.83	1.57	NS	NS
CD40LG	MA	−2.15	NS	NS	ND	ND	ND
	qPCR	−1.48	NS	NS	1.42	NS	NS
CX3CR1	MA	2.38	NS	−2.54	ND	ND	ND
	qPCR	−1.42	NS	−1.80	1.47	2.09	2.65
CXCL16	MA	−2.34	NS	2.13	ND	ND	ND
	qPCR	−1.23	NS	1.41	1.34	NS	NS
IFNGR2	MA	−2.34	NS	1.87	ND	ND	ND
	qPCR	−1.92	NS	1.56	3.53	2.26	NS
IL13RA1	MA	−2.19	−1.63	NS	ND	ND	ND
	qPCR	−2.79	−2.30	NS	3.93	3.24	NS
EGR1	MA	−2.21	NS	NS	ND	ND	ND
	qPCR	−1.26	NS	NS	−9.85	−10.15	−12.43
PPBP	MA	−3.82	NS	NS	ND	ND	ND
	qPCR	−3.18	NS	1.67	NS	NS	−3.81
KLF4	MA	−2.65	−1.92	NS	ND	ND	ND
	qPCR	NS	−1.23	NS	2.16	2.06	NS
OAS1	MA	1.74	NS	−2.35	ND	ND	ND
	qPCR	2.23	NS	NS	13.31	17.81	29.71
P2RY13	MA	−2.36	−2.38	NS	ND	ND	ND
	qPCR	NS	−2.30	NS	2.26	3.27	1.42
VCAM1	MA	2.46	NS	−2.78	ND	ND	ND
	qPCR	NS	NS	NS	−24.33	−13.22	−21.75
TNFRSF9	MA	2.07	NS	−2.40	ND	ND	ND
	qPCR	1.66	NS	−2.10	2.66	5.59	4.42
GPR56	MA	2.16	NS	−1.83	ND	ND	ND
	qPCR	3.45	NS	−1.71	17.74	30.27	61.20

**NOTE.** *vs*, versus; NS, not significantly changed; ND, not detected; qPCR, quantitative real-time polymerase chain reaction; MA, microarray analysis.

## Discussion

It is well-known that concomitant HIV infection could decrease the effects of anti-HCV treatment, and accelerate the progression of HCV-associated liver disease. On the other hand, accumulated evidence shows that concomitant HCV infection may also impair immune reconstitution and accelerate the AIDS progress. However, little is known about the molecular basis of HIV and HCV interactions and their modulations on host responses. Because CD8^+^ T cells play a crucial role in the eradication of viruses and infected target cells, for the first time we investigated the gene expression profiles of CD8^+^ T cells in treatment-naïve HCV, HIV-mono-infected and HIV/HCV-co-infected individuals.

In this study, we employed microarray assays to reveal the globally differentially expressed genes of CD8^+^ T cells between the HIV/HCV co-infected, the HCV and the HIV mono-infected groups. Our results indicated that the gene expression profile of CD8^+^ T cells in the HCV mono-infected groups differed drastically from that of the HIV mono-infected groups. GO analysis revealed that category of cell cycle was mainly and significantly altered between these two groups. It is interesting to note that a great number of genes, such as TPX2, CCNA2, KIF11, WEE1, ASPM, MKI67, and NUSAP1, which function mainly in mitosis of cell cycle progress, were significantly up-regulated in HIV infections. Together with genes important for DNA package such as HIST1H3B, HIST1H2AJ and HIST1H1B, the deregulated expressions of these genes may lead to disordered cell cycle progression[25], [26], [27], [28]. In HIV infections, high incidence of premature chromatid separation (PSC) occurred [29]. However, the required molecules remain to be clarified. Previous studies have reported that by displacing heterochromatin protein 1-α (HP1-α) and HP1-γ from chromatin, the Vpr protein could disrupt the higher order structures of heterochromatin, and thus lead to PSC in HIV-infected cells [30]. Our results indicate that genes regulating mitosis and the histones may also be possible targets for HIV virus to induce PSC.

The second characteristic pattern of the DE genes between these two groups is the enriched GO categories associated with cell migration. These categories included many cytokines in the enriched KEGG pathways. The proteins encoded by these genes are of particular interest because they are primarily responsible for regulating leukocyte traffic and play fundamental roles in homeostasis as well as other functions of the immune system. These data suggest a higher recruitment trend of blood CD8^+^ T cells to inflamed tissues (such as those in the liver) in HCV-infected patients, as indicated by strongly increased expressions of CCR4 and CXCL16 in HCV-mono-infected samples. Our results together with other studies highlighted the associations between chronic HCV infection and the increased infiltration of lymphocytes into the liver [31]. It is well-known that HCV could easily elude host immunity by induction of the T cell exhaustion [32], but the mechanisms are not yet understood. One study suggested that the inhibitory molecule programmed death-1 may be implicated in this process [33]. Our study was not only consistent with this finding, but identified many other genes in regulation of cell apoptosis as well. For example, HIPK2, a protein positive regulating cell death, showed a high expression level in HCV infections. This protein may promote CD8^+^ T cell death in a p53 dependent pathway, thereby leading to CD8^+^ T cell exhaustion during HCV infection. CD38, which functions as a suppressor of apoptosis, was down-regulated in HCV infected patients. As a marker of active CD8^+^ T cells [34], the decreased expression level of CD38 reflected the lower active CD8^+^ T cell status in the HCV mono-infected individuals. These results were highly consistent with the previous observations. Unlike HIV infections, CD8^+^ T cell responses in blood are very hard to detect in patients who are persistently infected with HCV[35]. However, some of the specific gene expression patterns, which were identified in the early and chronic HIV-1 infections [36], were not found in our study. For example, when comparing the HCV-mono-infected patients, we neither detected significant interferon response, nor identified downregulated receptors in the HIV-mono-infected patients. These phenomena indicate that although each virus displays a distinct pathogenesis, both HIV and HCV could create a state of super-infection immunity by modulating the interferon response and the downregulation of receptors.

To uncover the impact of HIV on the HCV-specific immune response, the gene profiles in the CD8^+^ T cells between the HIV/HCV co-infected and the HCV mono-infected groups were compared. Previous evidence suggested that immune suppression mediated by HIV infection was generally associated with a more rapid progression of HCV-related liver diseases [37]. Our results confirmed this hypothesis by providing direct evidence of gene expression profiles. Unlike a recent study conducted in liver biopsies, in which the global gene expression profiles in the HCV- and the HIV/HCV-co-infected individuals did not differ significantly [36], our results identified subsets of down-regulated genes in the HIV/HCV-co-infected group, compared to the HCV mono-infections. Among the total 55 DE genes between the two groups, more than 80% (47 genes) of them were down-regulated. GO analyses revealed that many of them were correlated with immune responses. For example, genes regulating interleukin-2 (IL-2) and genes responding to interferon-gamma showed a prominent decrease in the CD8 T cells in the HIV/HCV co-infected groups. Because the production of cytokines such as IL-2 and IFN-γ is characteristic of poly-functional T-cells, a decrease in their expression likely indicated the absence of polyfunctional T-cell responses in the HIV/HCV-co-infected individuals [38], [39]. Instead, HCV-specific T-cell responses in the co-infected patients showed an effector profile, which was associated with a significantly higher HCV viral load and more severe liver fibrosis scores [40]. In our study, we showed that not only a number of genes involved in T lymphocyte proliferation, activation and differentiation were down-regulated, but also that genes engaged in B cells activation were suppressed (such as MS4A1, CD40LG, and IL13RA1) in the co-infected individuals. These indicate the possibility that the B cell responses to HCV are also inhibited in the presence of HIV, and the impact of HIV on HCV-associated disease progression could occur through modulation of an immune response limited not only to the T cells. Besides, both the functional annotation and the KEGG pathway analysis showed suppressed genes expressions in lysosome, antigen processing and presentation pathways. These expression profiles offered partial explanations for immune suppression in the HIV/HCV co-infected patients [41]. We also noted a distinct expression pattern of cell adhesion molecules compared to those from liver biopsies. Our data illustrate a limitation in the recruitment of lymphocytes into the liver in co-infected individuals. Although it is difficult to predict the effect of this distinct phenomenon, one interpretation is that HIV may limit the tissue damage associated with lymphocyte infiltrates, it might exacerbate the harmful effects of HCV virus itself by preventing the major antiviral lymphocytes migrating to the liver.

The impact of HCV infection on HIV/AIDS is controversial [22]. Functional genomic analysis of CD8^+^ T cells demonstrated that the global gene expression profiles in the HIV- and the HIV/HCV-infected individuals did not differ significantly. This is consistent with a retrospective study in which the medical histories of 238 patients were analyzed but did not find a statistically significant difference in HIV disease progression in patients with an initial CD4 count above 600 [16]. The lack of significant difference in CD8^+^ T cell gene expression may suggest that the mechanism of HIV-associated immunological disease in HCV (−) and HCV (+) patients is similar. This may, however, only because of the weak blood CD8^+^ T cell response for HCV. Furthermore, the minor populations of CD8+ T cells programmed to control the virus were diluted out in the total T-cell populations examined. Besides, we also cannot ignore the fact that all of these participants are asymptomatic and treatment-naïve carriers. According to a previous study [23], these non-progressors with preserved T-cell functions usually showed T-cell gene expression profiles which were similar to those of uninfected individuals. However, despite the similarities between these two groups, some interesting genes still deserve attention. The GO analysis still identified some interesting genes. For instance, GPR15, which was up-regulated in the HIV/HCV co-infected patients, was initially discovered as a G-protein coupled receptor, and later found to be the co-receptor required for HIV-2 infection. The HIV-2 isolated from individuals with and without plasma viremia used this protein as one of the major co-receptors [48]. The increase in GPR15 expression may allow better entry and infection of HIV in the HIV/HCV co-infected patients than in the HIV mono-infected ones. Taken together, regarding the role of HCV in HIV-associated disease progression, investigations of other parts of cell populations such as CD4^+^ T cells and liver cells may provide compensatory information.

To validate our results, quantitative RT-PCR experiments were used. As shown in Figure 2 and Table 3, the results largely confirmed the data from the microarray assays. We also evaluated the expression levels of these selected genes in un-infected persons. According to our results, 7 genes showed similar expression patterns in the infected groups when compared to un-infected persons. 5 of them increased, 2 decreased in the infected groups. It is supposed that these two viruses may exert synergistic effects in co-infections. For instance, the expression level of EGR-1 significantly decreased in all the infected groups related to un-infections; while compared to the HCV infected groups, it decreased much more in the HIV/HCV co-infected groups. Considering that EGR-1 plays such important roles in the development and maturation of both the B and T cells, we believe that the down-regulation of EGR1 in HCV and/or HIV infected persons would significantly contribute to the disease progression. We also identified 3 genes only altered expressions in the HCV infected groups when compared to health controls. However, no significant variance was detected when compared their expressions levels between the HIV mono-infected groups to HIV/HCV co-infected groups. Combined with the little difference indentified in global gene expression comparison between these two groups, we postulate that the HCV infection may only have minimal effects on HIV CD8+ T cell gene expressions. For genes including: IFNGR2, IL13RA1 and KLF4, little changes were identified in co-infections when compared to un-infected persons. This seems some contradictory cause significant increased expressions were observed both in HCV and HIV mono-infections. To understand the real expression profiles, large sample screen may needed.

It was a challenge to get sufficient RNA from a single subject cell population for transcriptomics studies. In our study, the participants were divided into three groups (HIV, HCV-mono-infected, and HIV/HCV-co-infected). Equal amounts of CD8^+^ T cells from eight individual in the same group were pooled to form a subgroup or replicate. We successfully constructed three biological replicates in each group. It is suggested that pooling dramatically improves the accuracy in small designs in which no more than three arrays were available in each biological condition [42], and the more samples pooled, the greater was the improvement in statistical power [43]. Thus, the RNA pooling scheme used in this study not only provided adequate statistical power (as shown by qRT-PCR validation) but it also decreased the cost of the microarray experiments.

In summary, this is the first study to evaluate the global gene expression patterns of HIV/HCV co-infection against mono-infection in primary CD8^+^ T cells. Data analysis indicates that cellular processes in the HIV, HCV-mono-infected and the HIV/HCV-co-infected patients have indeed been altered. The alteration of genes, which play a crucial role in cell proliferation/activation/differentiation, and T cell regulation and cytokine responses have also been noted. The information presented here, therefore, not only lays down the basis for future studies, but also helps us to identify new markers for the management and treatment of HIV/HCV-co-infected patients.

## Materials and Methods

### Treatment-naïve HIV/HCV-mono-/co-infected individuals

During the initial step, more than 300 HIV-infected (45% with HCV co-infection) and 150 HCV-mono-infected individuals with informed consent were enrolled at the Shenzhen Center for Disease Control and Prevention. Written content was obtained from the participants. Ethics approval was obtained from The Joint Chinese University of Hong Kong–New Territories East Cluster Clinical Research Ethics Committee. All these participants were screened for HIV (Beijing Wantai Biological Pharmacy Enterprise Co. Ltd, Beijing), HCV (Beijing Wantai Biological Pharmacy Enterprise Co. Ltd, Beijing) and HBV (Beijing Wantai Biological Pharmacy Enterprise Co. Ltd, Beijing) antigens and antibodies by the standard ELISA analysis recommended by The Center of Disease Control and Prevention of China (guidelines of CDC, China). HIV infection was further confirmed by Western blot (HIV Blot 2.2 WB; Genelabs Diagnostics, Singapore) following the National Guideline for Detection of HIV/AIDS of China. The viral RNA were isolated and applied for RT-PCR, and the sequences of reverse-trancriptase (RT) and proteinase (P) genes were determined by automated sequencing the RT-PCR products. In order to eliminate the confounding factors, including treatment responses and drug-resistant bias, all HIV+ patients were tested for CD4 counts with flow cytometry, and for drug-resistant mutations by genotyping analysis. All our enrolled HIV+ individuals were antiretroviral-naïve with CD4 counts ≥300 cells/ µl and without baseline drug-resistant mutation. Based on the stringent criteria described above, 24 samples from each infected group were selected in this study. For the validation of our findings by quantitative real-time PCR, 24 healthy individuals were further enrolled. Their demographic and epidemiological data have been documented (Table S3).

### Purification of CD8^+^ T cells and RNA isolation

Peripheral blood mononuclear cells (PBMCs) were separated and purified immediately after the blood samples (30 mL whole blood from each individual) were obtained by Ficoll-Hypaque separation. Fresh CD8^+^ T cells were then obtained by positive isolation by the use of microbead immunoselection (Miltenyi Biotec, Oslo, Norway). To maximize the RNA yields and to minimize possible changes in the gene expression profiles, these aspects were strictly followed, as described in a previous study [44]. The purities of isolated CD8+ T cells were measured by Flow-cytometric analysis of cells markers (CD8). This demonstrated that 97.8%±0.020 (mean ± SD), 96.8%±0.023 (mean ± SD) and 97.8%±0.021 (mean ± SD) of purified CD8+ cells were single positive for the CD8 marker in the HIV-infected group, the HCV-infected group, and the HIV/HCV-co-infected group, respectively. For microarray analysis, the RNA pooling method was used to reduce the noise and to enhance the reliability when many subjects are pooled without the loss of individual specific information [43]. In each infection group, samples were divided into three subgroups for independent replicates. In each subgroup, equal amounts of CD8^+^ T cells from eight individuals were pooled for RNA isolation and the following transcriptomic studies. The left CD8^+^ T cells were stored in a freezer at −80^°^C for validation. RNA was isolated by using an RNAeasy Total RNA Isolation Kit (Qiagen, Germany), and it was then applied in microarray assays.

### Microarray assays

The total RNA pools were prepared for microarray analysis by using the Affymetrix GeneChip Human Gene 1.0 ST Array, which interrogates 28,869 genes across 764,885 distinct probes and contains more than 99% of the sequences present in the RefSeq database (Affymetrix, Santa Clara, CA). The Affymetrix GeneChip Whole Transcript Sense Target Labelling Assay Manual Version 4 was used for complementary DNA (cDNA) generation, hybridization, and array processing. Briefly, 300 ng total RNA underwent first-strand and second-strand cDNA synthesis. Complementary RNA was generated and used to produce sense-strand cDNA, which was fragmented and end-labelled with biotin. Biotin-labelled cDNA was hybridized to the Human Gene 1.0 ST Array for 16 hours at 45°C by using the GeneChip Hybridization Oven 640 (Affymetrix). Washing and staining with streptavidin-phycoerythrin was performed by using the GeneChip Fluidics Station 450, and images were acquired by using the Affymetrix Scanner 3000 7G Plus (Affymetrix). Following scanning, the CEL files were imported into Partek Genomic Suitev6.4 (Partek Inc, St Louis, MO), and the expression value for each probe set was calculated by using a robust multi-chip average (RMA) algorithm, as previously described [45]. A two-way Annova test was applied to identify differentially expressed (DE) genes (fold change >2 and *p value* < 0.05).

### Bioinformatic analysis

Differentially expressed genes in each comparison were annotated into biologically relevant categories using the web-based DAVID bioinformatics resources 6.7 (the Database for Annotation, Visualization, and Integrated Discovery, http://david.abcc.ncifcrf.gov/). For GO enrichment analysis, the minimum number of genes for corresponding term was set as 2. The threshold of the Expression Analysis Systematic Explorer (EASE) score, a modified Fisher exact p value, for gene-enrichment analysis was set at 0.05 (p≤0.05 is considered strongly enriched in the annotation categories). The enriched KEGG pathways were obtained from WebGestalt (Web-based Gene SeT AnaLysis Toolkit, http://bioinfo.vanderbilt.edu/wg2/). The hypergeometric test was used; all genes from humans were used as reference. KEGG pathways with at least two genes and *p*<0.01 are identified as enriched.

### Quantitative real-time PCR

The specific primers were designed by using Primer Express 3.0 software (Applied Biosystems) and tested for specificity by using NCBI's BLAST software. Primers used in this study are listed in Table S4. The quantitative real-time PCR (qRT-PCR) was carried out by using the ABI 7500 Real-Time PCR system with Power SYBR Green Master Mix (Applied Biosystems, USA) using a similar protocols, as previously described[46].

### Statistical analysis

Data were analyzed with SPSS version 13.0 (SPSS Inc., Chicago, IL, USA). A nonparametric test was used for pairwise comparisons. *p* value <0.05 was considered as statistically significant.

## Supporting Information

Table S1 **Differently expressed transcript identifiers in each paired comparisons.**
(XLS)

Table S2 **Significant gene ontology (GO) biological process terms identified in each paired comparisons.**
(XLS)

Table S3 **Participants clinical details.**
(XLS)

Table S4 **Primers used in the quantitative real time PCR.**
(PPT)
