# Supplementary material for: Transcriptomic Assay of CD8+ T Cells in Treatment-Naïve HIV, HCV-Mono-Infected and HIV/HCV-Co-Infected Chinese
Source: PLoS One. 2012 Sep 13;7(9):e45200. doi: 10.1371/journal.pone.0045200 (PMC3441577; doi:10.1371/journal.pone.0045200)
Supplement: Table S4 — Primers used in the quantitative real time PCR. (PPT) [file pone.0045200.s004.ppt]

## Slide 1
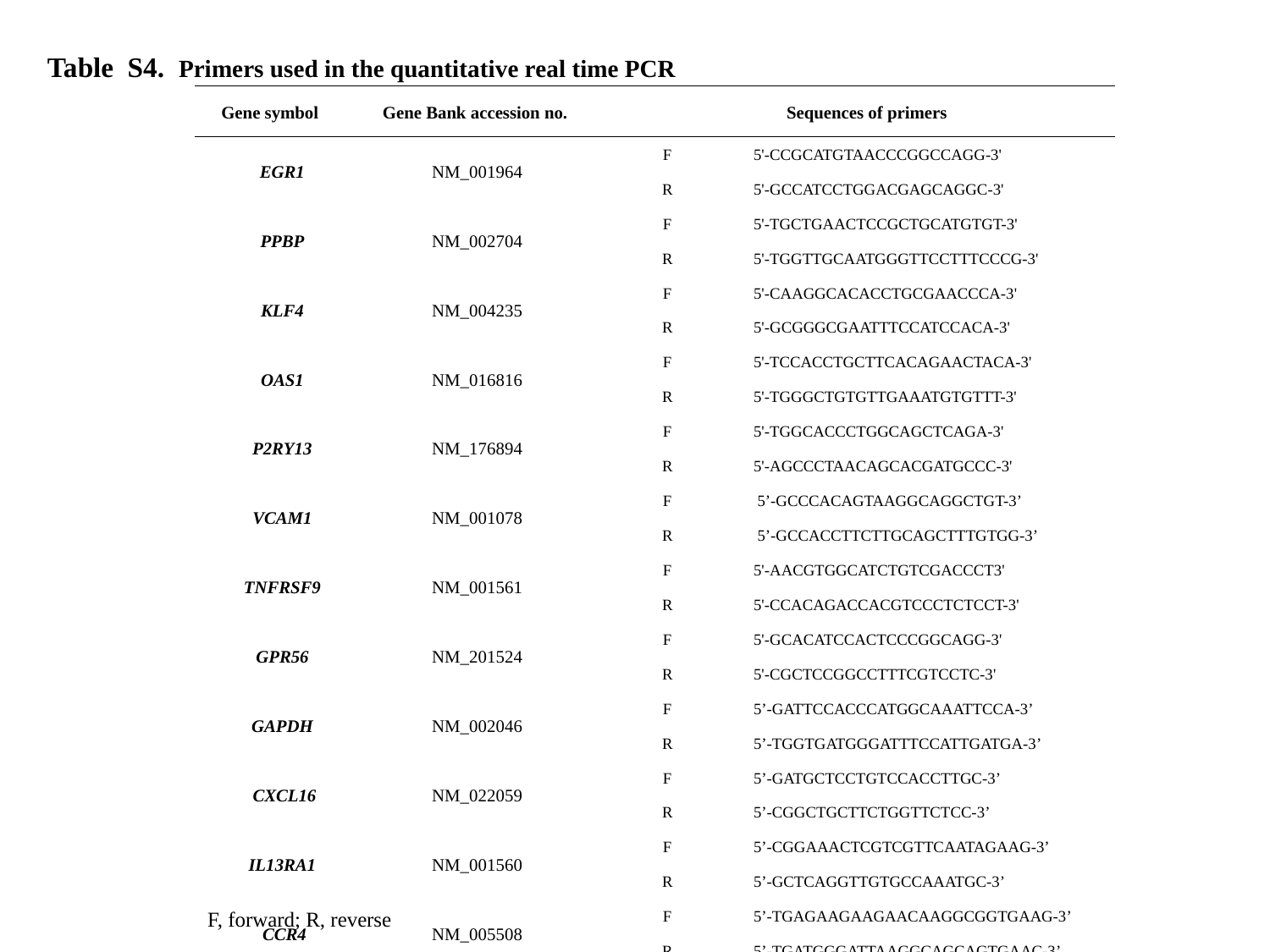

Table S4. Primers used in the quantitative real time PCR
| Gene symbol | Gene Bank accession no. | Sequences of primers | |
| --- | --- | --- | --- |
| EGR1 | NM\_001964 | F | 5'-CCGCATGTAACCCGGCCAGG-3' |
| | | R | 5'-GCCATCCTGGACGAGCAGGC-3' |
| PPBP | NM\_002704 | F | 5'-TGCTGAACTCCGCTGCATGTGT-3' |
| | | R | 5'-TGGTTGCAATGGGTTCCTTTCCCG-3' |
| KLF4 | NM\_004235 | F | 5'-CAAGGCACACCTGCGAACCCA-3' |
| | | R | 5'-GCGGGCGAATTTCCATCCACA-3' |
| OAS1 | NM\_016816 | F | 5'-TCCACCTGCTTCACAGAACTACA-3' |
| | | R | 5'-TGGGCTGTGTTGAAATGTGTTT-3' |
| P2RY13 | NM\_176894 | F | 5'-TGGCACCCTGGCAGCTCAGA-3' |
| | | R | 5'-AGCCCTAACAGCACGATGCCC-3' |
| VCAM1 | NM\_001078 | F | 5’-GCCCACAGTAAGGCAGGCTGT-3’ |
| | | R | 5’-GCCACCTTCTTGCAGCTTTGTGG-3’ |
| TNFRSF9 | NM\_001561 | F | 5'-AACGTGGCATCTGTCGACCCT3' |
| | | R | 5'-CCACAGACCACGTCCCTCTCCT-3' |
| GPR56 | NM\_201524 | F | 5'-GCACATCCACTCCCGGCAGG-3' |
| | | R | 5'-CGCTCCGGCCTTTCGTCCTC-3' |
| GAPDH | NM\_002046 | F | 5’-GATTCCACCCATGGCAAATTCCA-3’ |
| | | R | 5’-TGGTGATGGGATTTCCATTGATGA-3’ |
| CXCL16 | NM\_022059 | F | 5’-GATGCTCCTGTCCACCTTGC-3’ |
| | | R | 5’-CGGCTGCTTCTGGTTCTCC-3’ |
| IL13RA1 | NM\_001560 | F | 5’-CGGAAACTCGTCGTTCAATAGAAG-3’ |
| | | R | 5’-GCTCAGGTTGTGCCAAATGC-3’ |
| CCR4 | NM\_005508 | F | 5’-TGAGAAGAAGAACAAGGCGGTGAAG-3’ |
| | | R | 5’-TGATGGGATTAAGGCAGCAGTGAAC-3’ |
| IFNGR2 | NM\_005534 | F | 5’-CCGCCAGACCCTCTTTCC-3’ |
| | | R | 5’-GCCTCGTGCTATTGCTCAG-3’ |
| CD40LG | NM\_000074 | F | 5’-AGCCAGCCTCTGCCTAAAG-3’ |
| | | R | 5’-CACATTGACAAACACCGAAGC-3’ |
| CX3CR1 | NM\_001337 | F | 5’-CTTCATCACCGTCATCAG-3’ |
| | | R | 5’-GTAGTCACCAAGGCATTC-3’ |
F, forward; R, reverse
